# Gender-Based Differences in the Consumption of Food Rich in Fibre and Its Relationship with Perceived Mood Status: A Cross-Sectional Study

**DOI:** 10.3390/healthcare10040730

**Published:** 2022-04-14

**Authors:** Mudi H. Alharbi, Sehad N. Alarifi

**Affiliations:** 1 Clinical Nutrition Department, College of Applied Medical Sciences, Taibah University, Madinah 42353, Saudi Arabia; 2 Department of Food and Nutrition Science, Al-Quwayiyah College of Sciences and Humanities, Shaqra University, Shaqraa 11971, Saudi Arabia; snalarifi@su.edu.sa

**Keywords:** source of fibre, men and women, depression, anxiety, stress

## Abstract

It is unclear whether gender-based differences in dietary fibre intake exist in the relationship between daily fibre consumption and the prevalence of mood disorders. This study aims to examine the effects of dietary fibre consumption on mood status between genders in Saudi Arabia. A total of 359 Saudi participants completed the survey. The data showed that women consumed 14 g fibre/day and had a mild depression score, while men consumed 12 g/day and had very severe stress. The consumption of low-to-moderate servings of fruit or very low servings of nuts and seeds was associated with stress in men. Moderate levels of depression among women were likely to occur with low-to-moderate servings of nuts and seeds. Moderate-to-high stress levels among women appeared to be associated with low-to-moderate servings of vegetables, while depressed men consumed vegetables in low-to-moderate servings. However, anxious women who experienced mild levels consumed low-to-moderate servings of vegetables, and those with a mild-to-moderate level of anxiety consumed low-to-moderate servings of bread, whole grains, and cereals. The preliminary results showed that the consumption of 12 g fibre/day is not enough to relieve stress among men, while 14 g/day reduces the level of depression to mild among women. Fibre-rich foods, e.g., vegetables, nuts and seeds, fruit, bread, cereals and legumes, lower the degree of negative moods, but this is not only attributed to fibre, as there are other influential nutrients.

## 1. Introduction

A considerable body of literature has developed around the relationship between fibre and mental health; however, the mechanism of its influence on mental health is still unknown [1]. The definition of fibre is clarified by Stephen et al. [2] as “Carbohydrate polymers with three or more monomeric units (MU) that are neither digested nor absorbed in the human intestine, and it includes: (1) non-starch polysaccharides (NSP) from fruit, vegetables, cereals and tubers, whether intrinsic or extracted, chemically, physically and/or enzymically modified or synthetic (MU ≥ 10); (2) resistant (non-digestible) oligosaccharides (RO) (MU 3–9); and (3) resistant starch (RS) (MU ≥ 10)”. In general, wholegrain products and cereals, fruit, vegetables, legumes, seeds and nuts are considered as foods that are high in fibre. The recommended fibre intake for an adult is 25 g for women and 30–38 g/day for men (14 g per 1000 kcal per day) [3]. However, the diet of many of people in Middle Eastern countries is lacking in foods sufficient for the recommended consumption of fibre [4]. Additionally, evidence from previous studies indicates conflicting findings on gender-based differences in diet and nutritional intake [5,6]. Thus, this maybe highlights the gender differences in the consumption of fibre and the health consequences it may have. Accordingly, studies conducted in developing countries such as Bangladesh illustrate a reduced incidence of fibre intake in women, leading to a higher risk of nutrient deficiency and health-related problems compared to men [6]. However, the consumption of adequate amounts of fibre has several health benefits, including a lower incidence of mood disorders such as depression, stress and anxiety [7]. Lately, the importance of the correlation between dietary fibre intake and mood disorders in managing mental well-being has attracted considerable interest [7]. Indeed, epidemiological studies have demonstrated that adherence to healthy dietary patterns, including those with a high consumption of fibre, is associated with a lower risk of depression [8]. However, the nature of these correlations is complex due to the two-way causal effects between dietary fibre intake and mood disorders. For instance, changes in food preferences due to short-term mood status, such as eating comfort foods during mood swings or experiencing changes in appetite due to stress, are general human experiences. A more recent explanation for how fibre intake may affect mood status is the effect of dietary patterns on the gut microbiota: the human microbiota is increasingly recognized as a main factor impacting well-being and health, with diverse possible benefits including enhanced cognition and mood [9]. Recognizing the need for a better understanding of gender-based differences in the consumption of fibre and acknowledging the relationships between diet and mood status, this study aims to examine potential gender differences in the consumption of dietary fibre intake and whether and to what extent such differences affect perceived mood status in adult men and women in Saudi Arabia. A greater understanding of gender-based differences in dietary fibre intake would be useful in optimizing existing nutritional interventions and facilitating the execution of new comprehensive interventions intended to enhance the nutritional status and health profile of populations [5].

## 2. Materials and Methods

### 2.1. Design and Study Participants

The present study, a quantitative cross-sectional exploratory study in Al-madinah Al-munawwarah and surrounding areas in Saudi Arabia, was started in November 2018 and terminated in June 2019. Eligible participants for this study were identified using a health and lifestyle questionnaire. Advertisements on social media and flyers on university campuses were also employed as recruitment strategies. The inclusion criteria for the target population for the present study were as follows: healthy adults aged ≥19 to ≤45 years with a body mass index (BMI) >18.5 and <25 kg/m^2^. The exclusion criteria were pregnancy and breastfeeding; premenstrual syndrome (PMS); considerable incidents such as weight loss, weight gain or dietary change within the past six months; suffering from food intolerances such as lactose intolerance or gluten sensitivity; suffering from disorders such as adverse gastrointestinal symptoms, depression, or cardiovascular or metabolic diseases; continuous long-term consumption of corticosteroid drugs; using fibre or supplements, e.g., probiotics or/and prebiotics; and smoking cigarettes more than 14 times per week or shisha or hookah more than once per week. In total, 390 participants were invited to take part in the study, of whom 31 were removed. Finally, after excluding respondents who did not meet the criteria, 359 participants (179 men and 180 women) were included for data analysis, with a response rate of 92.05%. A sample size of 359 provides a power of at least 94%, with a margin of error of 5%. Participation in the study was voluntary and anonymous. Participants gave their informed consent and then completed the questionnaire. Questionnaires were distributed to the participants by email and/or WhatsApp. The main questionnaire included all the following in one form: a consent form, a health and lifestyle questionnaire (for screening and obtaining information about confounding variables), a 24 h dietary recall form (dietary intake and energy intake per day), a food frequency questionnaire (FFQ) (usual intake during the last six months) and a mood test, which were filled out one at time. Each participant provided signed informed consent before participating in the study, which was conducted in accordance with the Declaration of Helsinki. This study was ethically approved by Taibah University (no CLN201806)

#### 2.1.1. Data Collection Measures

##### Demographic and Anthropometric Data

Gender, marital status, income, education level and age were recorded in the demographics, and BMIs were recorded as the anthropometrics, as well as smoking status. All this information was obtained from the health and lifestyle questionnaire, and the energy intake per day was also recorded from 24 h dietary recall.

##### Dietary Assessment Methods

This study adopted an FFQ [10] and a 24 h dietary recall to assess the consumption of dietary fibre in the target populations. The semi-quantitative FFQ questionnaire concentrated on usual consumption during the last six months (on average). It measured the consumption of some major fibre-containing food items such as cereals and bread, vegetables and fruit, nuts and seeds, and legumes. The participants were asked how often they consumed certain food products. Consumption was divided into three groups: (1) no consumption or very low consumption, (2) low-to-mild consumption, and (3) moderate-to-high consumption.

Each response on the frequency of food consumption was inserted under one of the three main categories as follows: (1) no consumption or very low consumption: responses of “never or less than once a month” and “one to three servings a month”; (2) low-to-moderate consumption: responses of “once a week” and “two to four servings a week”; (3) moderate-to-high consumption: responses of “five to six times a week”, “once a day”, “two to three servings a day”, “four to five servings a day” and “six plus times a day”.

A 24 h dietary recall was obtained from the participants, who were asked to recall all food items and beverages consumed from waking up until going to sleep the previous day. The information requested included estimates of the amount consumed of food items that contained fibre, such as cereals, wholegrain products, fruit and vegetables, nuts; pulses such as beans, lentils or chickpeas; and salads. The average amount of each food item was determined in relation to commonly used household utensils and with a handy guide to portion sizes, with photographs that indicated different portion sizes of certain food items. This enabled participants to make more accurate estimates of the quantities of food consumed, minimizing errors in estimation. These results were analysed using the software Dietplan7 (Forestfield Software Ltd, Horsham, UK). 

##### Depression Anxiety Stress Scales (DASS) Questionnaire

The prevalence and severity of the negative mood statuses of the participants were assessed using the 21-item self-administered Depression Anxiety Stress Scales (DASS-21) questionnaire with seven categories per subscale [11], which has been validated for non-clinical use in populations and research [12], including in the Arabic language [13]. Each of the questions is scored from 0 (did not apply to me at all) to 3 (applied to me very much), resulting in a total score of 0 to 21. In relation to symptoms of depression, participants with total scores of 0–4 were classified as normal, 5–6 as mild, 7–10 as moderate, 11–13 as severe and >14 as very severe. The scores for anxiety were 0–3 (normal), 4–5 (mild), 6–7 (moderate), 8–9 (severe) and >10 (very severe), and for stress 0–7 (normal), 8–9 (mild), 10–12 (moderate), 13–16 (severe) and >17 (very severe), respectively [11].

##### Statistical Analysis

All statistical analyses were carried out using the Statistical Package for Social Sciences (SPSS version 24.0 (IBM, Armonk, NY, USA)). Values are exhibited as mean ± standard deviation (SD) for continuous variables with *t*-testing to find the differences between genders, and as percentages for qualitative variables with chi-squared testing to find differences between genders. Descriptive statistics were evaluated for all parameters. The significance of differences in dietary fibre intake between men and women was analysed using the Kruskal–Wallis H test. Logistic regression was used to find the relationship between dietary fibre intake and perceived mood status (depression, anxiety and stress). In the analysis, all the potential confounding variables was analysed (Table S1) and with a significant association were included. Odds ratios (OR) calculated with 95% confidence intervals (CI) were calculated for both crude and adjusted data. One by one, variables with a *p* ≥ 0.15 have been excluded from the model by Wald statistic (backward method). The goodness of fit test was used to verify the model. The results are presented as odds ratios that are adjusted for confounding variables and accepted as significant at the level of *p* < 0.05. Mood was categorized into five levels: normal (treated as the reference category), mild, moderate, severe and extremely severe. Food consumption was divided into three levels: (1) no consumption or very low consumption, (2) low-to-mild consumption, and (3) moderate-to-high consumption.

## 3. Results

### 3.1. General Characteristics of the Study Population and Food Consumption

The demographic and anthropometric characteristics of the study population by gender are presented in Table 1. Of the 359 participants, 180 (50.1%) were women and the remainder were men (49.8%). The mean ages for men and women were similar. Both men and women had normal BMI, whereby men had a slightly higher mean BMI than women. The mean energy intake for men was higher than that of women participants. The mood test showed non-significant differences between both genders. The comparison between men and women in the study population showed significant differences in BMI, energy intake and smoking (*p* < 0.05), but not in age, income, education level and marital status (*p* > 0.05). However, the mean dietary fibre intake (according to the 24 h dietary recall) was significantly higher among women (14.0 ± 1.19) than men (12.0 ± 1.16).

### 3.2. Gender Differences in Consumption

Table 2 shows the frequency of consumption by men and women of each of the different food groups that contain fibre. There were significant differences in the intake of vegetables, bread and cereals, nuts and seeds, and legumes, but not fruit. In more detail, there were significant differences between men and women in terms of vegetable consumption (*p* < 0.05). A significantly greater percentage of men consumed low-to-moderate servings of vegetables (≤4 times/week) (72.6%), while 52.22% of women consumed a moderate-to-high servings of vegetables (≥once a day or 5 to 6 times per week), significantly more than the number of men who consumed moderate-to-high serving of vegetables (25.7%). In addition, a significantly greater percentage of women consumed low-to-moderate servings (≤4 servings/week) of bread, whole grains and cereals than men (women: 72.22%, men: 36.9%), albeit with men having a significantly moderate-to-higher consumption of bread, whole grains and cereals (≥once a day or 5 to 6 times per week) than women (men: 54.7%, women: 23.34%, *p* < 0.05). Additionally, men have a significantly greater moderate-to-high consumption of legumes than women (men: 44.13%, women: 35.56%, *p* < 0.05). However, a greater percentage of the study population, either men or women, consumed low-to-moderate servings of fruit (≤4 serving/week), with no significant differences between them (women: 69.4%, men: 67%, *p* > 0.05). Furthermore, a significantly higher percentage of women consumed moderate-to-high servings of nuts and seeds (≥once a day or 5 to 6 times per week) (54.44%), while a higher percentage of men consumed low-to-moderate servings of nuts and seeds (≤4 servings/week) (men: 49.2%, women: 25%, *p* < 0.05).

### 3.3. Relationship between Dietary Fibre Intake and Mood Status of the Study Population by Gender

Table 3 displays the logistic regression analysis of the significant relationship between mood status (stress, anxiety and depression) and a selection of various foods that are rich in fibre by gender, while controlling for potential confounding factors. See Table S2A&B (supplementary material) for the logistic regression analysis. In Table 3, women who experienced moderate (OR = 0.52, *p* = 0.025) to high (OR = 0.41, *p* = 0.006) stress levels were more likely to eat low-to-moderate servings of vegetables (≤4 serving) compared to unstressed women. The stressed men were likely to consume low-to-moderate servings of fruit (OR = 0.70, *p* = 0.017) or a very low serving of nuts and seeds (OR = 0.67, *p* = 0.049) (1–3 servings/month), or consumption total dietary fibre of ≤12 g per day (OR = 1.36, *p* = 0.033). Women who experienced mild levels of anxiety (OR = 0.60, *p* = 0.001) consumed low-to-moderate vegetable servings (≤4 serving/week), and more of those with mild-to-moderate anxiety consumed low-to-moderate servings of bread, whole grains and cereals (≤4 serving/week) than the non-anxious women (mild: OR = 0.65, *p* = 0.002, moderate: OR = 0.61, *p* = 0.011). Men who consumed low-to-moderate servings of vegetables (≤4 times per week) appeared to be more depressed (OR = 1.64, *p* = 0.038). Women who experienced moderate levels of depression were likely to consume low-to-moderate servings of nuts (≤4 serving/week) (OR = 0.81, *p* = 0.025), and a mild level of depression was associated with insufficient fibre intake per day (≤14 g; OR = 0.83, *p* = 0.043).

## 4. Discussion

The present study investigated gender-based differences in dietary fibre intake and the prevalence and severity of negative mood statuses (stress, anxiety and depression) between men and women in Saudi Arabia. The study observed a significantly higher intake of dietary fibre in women compared to men *p* < 0.05, in particular, a higher intake of vegetables, nuts and seeds, and total fibre per day (14 g) in women, although neither gender’s fibre consumption met the recommended daily fibre intake in adults, which is 25 g per day for women and 30–38 g per day for men [14]. Another important finding was that men showed a higher intake of bread, whole grains, and cereals than women, whereas there was a higher consumption of legumes by both genders. There was a similar consumption of fruit by both genders. The outcomes showed that women who consumed vegetables in low-to-moderate servings (*≤*4 serving per week) experienced moderate-to-severe stress. A similar finding was reported by Stephen et al. [15], who found that vegetable consumption was lower among stressed female students than male students. Additionally, Fernstrand et al. [16] demonstrated that greater perceived stress was associated with decreased vegetable consumption. Moreover, women in the present study experienced mild-to-moderate anxiety levels with low-to-moderate intake of bread, whole grains and cereals of less than or equal to four times per week. Additionally, they experienced mild anxiety with low to moderate intake of vegetables of less than or equal to four servings per week. These results are similar to those of Abbassi-Ghanavati et al. [17], who found that a higher intake of vegetables and whole grains is associated with decreased levels of anxiety and depression.

The scores show that women experience moderate depression with a low-to-moderate intake of nuts and seeds (*≤*4 serving per week). This echoes a study conducted by Arab et al. [18], which concluded that depression scores significantly decrease among nut consumers compared to non-nut consumers.

Female participants experienced mild depression with the consumption of 14 g of fibre per day. Despite this being less than the recommended total fibre intake per day for women, 14 g of fibre daily still lowered the level of depression experienced, and this needs to be investigated further. In addition, Xu et al. [19] found that 21 g per day of dietary fibre intake lowered the risk and severity of depression. However, in the present study, men experienced extremely severe stress with a consumption of 12 g of fibre per day, and the level of stress could be lowered by increasing the amount of fibre per day to, for example, 14 g or 21 g, so this needs to be investigated further. Grunberg and Straub [20] found that stressed men ate less fibre than non-stressed men, which could illustrate a decreased intake of healthy food and fibre among stressed men. The present study observed no association between total dietary fibre intake and depression in men with 12 g of intake, in contrast to the association in women with 14 g, which was significant. Additionally, in terms of stress, an association was observed between dietary fibre intake (12 g) and extremely severe stress in men, which was not the case in women (14 g). This could lead to the following conclusion: daily consumption of at least 14 g of fibre could perhaps help to decrease the level of depression among women, but not stress. With men, the opposite is the case: daily consumption of at least 12 g of fibre is not enough to reduce the level of stress. Several studies have reported an inverse relationship between depression and high consumption of fibre-rich foods such as vegetables, legumes, fruit and nuts [21,22].

Dietary fibre intake in the present study ranged between 12 g and 14 g per day, highlighting a gap between the current intake levels of the study participants and the recommended levels (25–38 g/day) in all age groups and in both men and women [3]. Similar findings were reported in a previous study where dietary fibre intake was reported to be below the recommended levels in men and women [3]. The observed gender differences may be attributed to the consumption of fibre from different dietary sources. A common concern in dietary studies that administer self-report questionnaires is the issue of cautious or unconscious underestimation [23]. This, to some extent, might explain the observed extremely low fibre intake in both men and women. In contrast to the present findings, a recent study by Abassi et al. [24] did not report any significant differences in dietary fibre intake between men and women in Tunisia. However, the present findings agree with a previous study by Kiefer et al. [25], where females (children, adolescents and adults) were inclined to consume more dietary fibre than males. This could be attributed to the more health-conscious nature of women and their tendency to comply with dietary recommendations more than men [25]. However, again in contrast to the present findings, a study by Bennett et al. [5] showed that men tend to have a higher fibre intake than women in the United Kingdom (UK).

A finding of the present study is that high consumption of vegetables, fruit, bread and cereals, nuts and seeds, and legumes was correlated with lower levels of stress, anxiety, and depression. These food groups are rich in fibre, omega-3 fatty acids, folate, tryptophan, magnesium, phytochemicals, and inositol. In fact, the full effects of fibre may not yet be recognized, and the overall effect is not attributable to it. Previous studies have documented a positive effect of these food groups on individuals’ mental well-being [26,27]. In addition, the present study observed that low consumption of vegetables, fruit, nuts, seeds and legumes was positively associated with stress, anxiety and depression. It is possible, therefore, that altered brain–gut interactions play a role in the mood status; for instance, changes in the psychological state of rodents after changes in their gut microbiota suggest the correlation of a depression-like state in humans with adverse changes in the gut microbiota [28,29]. The hypothesis that the consumption of fibre could affect the mood status seems to be a good fit given the relatively rapid effect of diet on mood disorders observed in humans [8], which follows a two-way interaction with the brain using neural, hormonal and inflammatory signalling [30].

Fibre-rich foods are considered to transform the gut microbiota and affect the brain function and mood status [8]. Remarkably, gender disparities in the impacts of dietary fibre intake on gut microbiota have been observed in humans, with a trend for larger dietary effects in men than in women. Additionally, susceptibility to mood disorders differs between the genders [31]. Hence, microbial differentiations could be the underlying reasons for gender differences in the relationship between dietary fibre intake and the mood status [3].

The present study has various strong points. First, this study was conducted with a representative sample of men and women covering a wide age range, allowing gender-based differences in dietary fibre intake to be examined. Second, the investigation of dietary fibre intake was based on the collection of an FFQ questionnaire and a 24 h dietary fibre assessment, which may ensure that the data collected are representative of fibre intake at the individual level. The dietary fibre intake information collected by the 24 h assessment approach is considered to improve the accuracy of fibre intake estimates in adults.

However, this study is not without limitations, and the present findings must be considered in light of the following. In this study, there were no laboratory assessments of dietary fibre intake. Assessment of the prevalence of depression, anxiety and stress was based on self-reporting, which might have led to recall bias and could necessitate a larger sample size. Given this, using another tool in addition to the DASS to assess the mood status might be more effective. Moreover, the cross-sectional nature of the present study does not indicate the causal effects and mechanisms underlying the potential effects of dietary fibre intake on depression and stress, and these need to be investigated in future studies. The limitations of the present study necessitate future research to further assess the potential effects of gender differences, especially in terms of fibre-rich food consumption and the real effect of fibre without an overlap with other nutrients. Although the present findings revealed that lower consumption of fibre-rich food items per day is associated with stress and depression, further studies are needed to determine the possible beneficial effect of specific amounts of fibre per day on the prevalence and severity of stress and depression. The integration of subjective assessment with objective assessment, including laboratory assessment of dietary fibre intake, is necessary to further explore its relationship with mood status.

Of interest in this study is the fact that gender-based differences in dietary fibre intake were noticeably associated with the prevalence of very severe stress in men and a mild level of depression in women during their adult years. These findings call for concerted dietary interventions aimed at improving nutrition and mood status for men and women, putting more emphasis on the quality of the fibre-rich foods consumed, in addition to their quantity, as this would lay the foundation for the empowerment of such interventions as well as the soundness of people in the future. Furthermore, the present outcomes have also highlighted higher intakes of dietary fibre in women compared to men, thus highlighting the need for interventions within particular and clear contexts pointed toward advancing better dietary habits in the Saudi population.

## 5. Conclusions

Taken together, the findings from the present study show that women consumed more fibre than men, even though their consumption of fibre did not meet the recommended level. However, 14 g of fibre per day may help to lower the depression score to mild among women, while 12 g of fibre per day may not lower the degree of stress among men. Additionally, regular consumption of foods that are rich in fibre, moderate-to-high servings (more than four times per week) of vegetables, nuts and seeds, fruit, bread, cereals and legumes may contribute significantly to decreasing levels of depression, anxiety and stress in men and women. However, this study cannot base the effect of fibre-rich food only on fibre, but there may be a contribution of other nutrients to this effect. Despite the study limitations, this study has clarified and suggested the association between dietary fibre intake and the prevalence of depression, anxiety and stress in Saudi women and men, as well as the associated gender-based differences. These findings also highlight the importance of the sources of dietary fibre as a factor influencing mood disorders. Therefore, future studies should confirm the effects of different doses of fibre and also the effect of different types of dietary fibre from different sources on mood disorders.

## Figures and Tables

**Table 1:** Characteristics of demographic, anthropometric, mood test and food consumption by gender of the total study population.

Particulars	Men	Women
	Mean (SD)	Mean (SD)
**Age (years)**	23.6 (5.54)	23.2 (5.21)
BMI *	23.47 (2.28)	22.31 (2.17)
**Marital Status (%)**	**n (%)**	**n (%)**
Married	89 (49.7)	81 (45)
Unmarried	90 (50.3)	99 (55)
**Income (SAR/month) (%)**	**n (%)**	**n (%)**
Low (<5000)	88 (49.16)	90 (50)
Average (5000–10,000)	41 (22.91)	43 (23.89)
Moderate (10,001–15,000)	30 (16.76)	28 (15.56)
High (>15,000)	20 (11.17)	19 (10.55)
**Education Level (%)**	**n (%)**	**n (%)**
High school	79 (44.13)	80 (44.44)
Diploma	14 (7.82)	12 (6.67)
Graduate	62 (34.64)	66 (36.67)
Post-Graduate	24 (13.41)	22 (12.22)
**Physical Activity (%)**	**n (%)**	**n (%)**
Yes	112 (62.6)	108 (60)
No	67 (37.4)	72 (40)
Smoking or/and tobacco products (%) *	**n (%)**	**n (%)**
Yes	120 (67)	55 (31)
No	59 (33)	125 (69)
**Supplements (%) ^$^**	**n (%)**	**n (%)**
Yes	36 (20)	42 (23)
No	143 (80)	138 (77)
Energy intake, mean (SD) ^!^*	1379.12 (242.34)	1181.35 (227.09)
Total Daily fibre intake, mean (SD) ^!^*	12.44 (2.88)	14.17 (3.09)
**DASS-Stress score** mean (SD)	11.15 (9.61)	12.86 (11.31)
**DASS-Anxiety score** mean (SD)	11.25 (9.09)	10.78 (9.78)
**DASS-Depression score** mean (SD)	14.11 (10.31)	12.32 (9.55)

SAR: Saudi Arabia Riyal, * trend *p*-value indicates statistical significance < 0.05, ^!^ from 24 dietary recall, $ supplements: minerals and vitamins, n; total number of participants.

**Table 2:** Frequency of fibre-rich food consumption according to gender.

Fibre-Rich Food	Men	Women
Vegetables *	**n (%)**	**n (%)**
No consumption or very low consumption	3 (1.7%)	2 (1.1%)
Low-to-moderate consumption	130 (72.6%)	84 (46.7%)
Moderate-to-high consumption	46 (25.7%)	94 (52.2%)
**Fruit**		
No consumption or very low consumption	10 (5.6%)	5 (2.8%)
Low-to-moderate consumption	120 (67%)	125 (69.4%)
High consumption	49 (27.4%)	50 (27.8%)
**Bread, whole grains and cereals**		
No consumption or very low consumption	15 (8.4)	8 (4.44)
Low-to-moderate consumption	66 (36.9%)	130 (72.22%)
Moderate-to-high consumption	98 (54.7%)	42 (23.34%)
Nuts and seeds *		
No consumption or very low consumption	36 (20.1%)	37 (20.56%)
Low-to-moderate consumption	88 (49.2%)	45 (25%)
Moderate-to-high consumption	55 (30.7%)	98 (54.44%)
Legumes *		
No consumption or very low consumption	26 (14.53%)	58 (32.22%)
Low-to-moderate consumption	74 (41.34%)	58 (32.22%)
Moderate-to-high consumption	79 (44.13%)	64 (35.56%)

* trend *p*-value indicates statistical significance < 0.05.

**Table 3:** Logistic regression analysis for significant relationship between fibre-rich food consumption and mood status among gender.

Food Category	Stress Adj.OR (95%CI)	*p*-Value	Anxiety Adj.OR (95%CI)	*p*-Value	Depression Adj.OR (95%CI)	*p*-Value
**Vegetables**	0.52 (0.04–0.69) ^$2^ 0.41 (0.01–0.53) ^$4^	0.025 0.006	0.60 (0.45–0.82) ^$1^	0.001	1.64 (1.03–2.62) ^!3^	0.038
**Fruit**	0.70 (0.01–1.41) ^!1^	0.017				
**Cereals, bread, whole grains**			0.56 (0.39–0.81) ^$1^ 0.61 (0.42–0.89) ^$2^	0.002 0.011		
**Nuts and seeds**	0.67 (0.45–1.99) ^!4^	0.049			0.81 (0.68–0.97) ^$2^	0.025
**Legumes**						
**Daily fibre intake ^+^ (g/day)**	1.36 (1.02–1.79) ^!4^	0.03			0.83 (0.69–0.99) ^$1^	0.043

^$^ female, ^!^ male, ^1^ mild effect, ^2^ moderate effect, ^3^ severe effect, ^4^ extremely severe effect, logistic regression with adjusted for BMI, smoking and energy intake, + obtained from 24 dietary recall, OR, odd ratio; CI, confidence interval.

## Data Availability

Not applicable.

## Supplementary Materials

The following supporting information can be downloaded at: https://www.mdpi.com/article/10.3390/healthcare10040730/s1, Table S1: Logistic regression analysis for the relationship between fiber-rich food consumption and mood status among women-model1. Table S2 A& B: Logistic regression analysis for relationship between fibre-rich food consumption and mood status among gender.
