# Supplementary material for: Gender-Based Differences in the Consumption of Food Rich in Fibre and Its Relationship with Perceived Mood Status: A Cross-Sectional Study
Source: Healthcare (Basel). 2022 Apr 14;10(4):730. doi: 10.3390/healthcare10040730 (PMC9030175; doi:10.3390/healthcare10040730)
Supplement: Supplementary file 1 [file healthcare-10-00730-s001.zip › healthcare-1644742-supplementary.pdf]

healthcare

MDPI

Table S1. Logistic regression analysis for the relationship between fiber-rich food consumption and mood status among women-model1.

Food Category	Stress OR (95%CI)	*p*-Value	Anxiety OR (95%CI)	*p*-Value	Depression OR (95%CI)	*p*-Value
**Vegetables**	1.53 (1.11–2.133) [$2]<br>1.55 (1.12–2.14) [$4]	0.040<br>0.037	1.17 (0.45–0.82) [$1]	0.045	1.32(1.11–2.67) [!3]	0.040
**Fruit**	0.88 (0.68–0.97) [!1]	0.035				
**Cereals, Bread, whole grain**			0.67 (0.42–0.89) [$1]<br>1.28(1.04–1.59) [$2]	0.043<br>0.052		
**Nuts and seeds**	0.89 (0.68–0.97) [!4]	0.052			1.37 (1.03–1.84) [$2]	0.042
**Legumes**						
**Daily fibre intake** [+] **(grams/day)**	1.63 (1.09–2.43) [!4]	0.042			1.64 (1.03–2.62) [$1]	0.053

$ female, ! male, [1] mild effect, [2] moderate effect, [3] severe effect, [4] extremely severe effect, logistic regression with adjusted all covaries, + obtained from 24 dietary recall.

Table S2. A: Logistic regression analysis for the relationship between fiber-rich food consumption and mood status among women.

Food category	**Women- adjusted OR (95%CI)**							
	Mild OR (**95%CI)**	P-value	Moderate OR (**95%CI)**	P-value	Severe OR (**95%CI)**	P-value	Extremely Severe OR (**95%CI)**	P-value
**DASS-STRESS**								
Vegetables	0.33(0.17-6.82)	0.47	**0.52(0.04-0.69)**	**0.02***	1.04(0.69-1.57)	0.85	**0.41(0.01-0.53)**	**0.006***
Fruit	0.96(0.70-1.32)	0.82	1.18(0.83-1.67)	0.36	1.08(0.74-1.57)	0.69	0.93(0.66-1.31)	0.69
Cereals, Bread, whole grain	0.75(0.52-1.10)	0.14	1.24(0.83-1.86)	0.30	1.21(0.79-1.86)	0.38	1.11(0.75-1.64)	0.61
Nuts and seeds	0.99(0.84-1.18)	0.94	0.60 (0.075-4.87)	0.64	1.03(0.84-1.27)	0.76	1.03(0.86-1.24)	0.74
Legumes	1.11(0.93-1.33)	0.26	1.06(0.85-1.32)	0.62	1.05(0.84-1.32)	0.64	1.06(0.87-1.29)	0.54
Daily fiber intake	0.98(0.85-1.12)	0.76	0.93(0.78-1.09)	0.38	1.01(0.85-1.19)	0.94	0.98(0.84-1.13)	0.75
**DASS-Anxiety**								
Vegetables	**0.60(0.45-0.82)**	**0.001****	1.50(0.72-3.15)	0.28	0.75(0.55-1.02)	0.07	0.81(0.54-1.21)	0.29
Fruit	1.03(0.76-1.40)	0.82	1.01(0.41-2.48)	0.98	1.03(0.76-1.38)	0.87	1.11(0.74-1.68)	0.61
Cereals, Bread, whole grain	**0.56(0.39-0.81)**	**0.002****	**0.61(0.42-0.89)**	**0.01***	1.46(0.51-4.23)	0.48	0.84(0.50-1.41)	0.52
Nuts and seeds	1.11(0.94-1.31)	0.23	1.92(1.03-3.58)	0.06	1.04(0.87-1.23)	0.61	0.87(0.68-1.09)	0.23
Legumes	0.93(0.78-1.12)	0.45	0.63(0.35-1.12)	0.12	0.96(0.79-1.16)	0.67	0.98(0.77-1.25)	0.89
Daily fiber intake	1.09(0.96-1.25)	0.19	1.14(0.81-1.62)	0.45	1.02(0.89-1.18)	0.76	1.12(0.93-1.35)	0.22
**DASS-Depression**								
Vegetables	1.51(0.98-2.31)	0.06	1.33(0.89-1.97)	0.15	1.39(0.93-2.07)	0.10	1.33(0.90-1.96)	0.15
Fruit	1.04(0.69-1.57)	0.85	1.21(0.84-1.74)	0.31	1.15(0.78-1.68)	0.48	1.18(0.82-1.72)	0.37
Cereals, Bread, whole grain	1.16(0.72-1.87)	0.54	1.16(0.75-1.80)	0.51	1.32(0.84-2.06)	0.22	1.34(0.87-2.06)	0.18
Nuts and seeds	1.09(0.87-1.37)	0.44	**0.81(0.68-0.97)**	**0.02***	0.40(0.03-0.54)	0.06	1.16(0.94-1.42)	0.17
Legumes	0.78(0.60-1.02)	0.07	1.10(0.88-1.38)	0.39	0.99(0.87-1.26)	0.95	0.97(0.77-1.22)	0.80
**Daily fiber intake**	**0.83(0.69-0.99)**	**0.04***	0.96(0.81-1.14)	0.65	1.11(0.93-1.33)	0.24	1.06(0.88-1.25)	0.54

Table S2. B: Logistic regression analysis for the relationship between fiber-rich food consumption and mood status among men.

Food category	Male adjusted OR (95%CI)							
	Mild OR (**95%CI)**	P-value	Moderate OR (**95%CI)**	P-value	Severe OR (**95%CI)**	P-value	Extremely Severe OR (**95%CI)**	P-value
**DASS-STRESS**								
Vegetables	1.41(0.86-2.31)	0.17	1.21(0.72-2.04)	0.47	1.29(0.77-2.18)	0.33	0.68(0.36-1.31)	0.25
Fruit	**0.70(0.01-1.41)**	**0.02***	1.32(0.79-2.19)	0.28	1.20(0.72-1.99)	0.49	0.87(0.47-1.60)	0.65
Cereals, Bread, whole grain	1.10(0.72-1.69)	0.65	1.25(0.79-1.97)	0.33	0.97(0.62-1.52)	0.89	0.88(0.51-1.53)	0.66
Nuts and seeds	1.03(0.77-1.37)	0.83	1.06(0.79-1.43)	0.68	1.04(0.77-1.41)	0.80	**0.67(0.45-1.99)**	**0.04***
Legumes	0.99(0.73-1.34)	0.94	1.07(0.78-1.48)	0.65	1.08(0.78-1.49)	0.65	0.89(0.61-1.31)	0.55
Daily fiber intake+	1.07(0.85-1.33)	0.57	1.13(0.90-1.43)	0.28	1.03(0.81-1.30)	0.82	**1.36(1.02-1.79)**	**0.03***
**DASS-Anxiety**								
Vegetables	1.43(1.03-1.98)	0.33	1.66(0.91-3.02)	0.09	1.63(1.09-2.43)	0.17	1.59(0.97-2.60)	0.06
Fruit	1.05(0.76-1.46)	0.74	0.74(0.41-1.33)	0.31	1.29(0.87-1.93)	0.19	1.32(0.81-2.16)	0.27
Cereals, Bread, whole grain	1.34(1.00-1.80)	0.49	1.51(0.87-2.62)	0.15	1.43(1.00-2.04)	0.15	1.37(0.89-2.09)	0.14
Nuts and seeds	0.95(0.79-1.15)	0.63	0.97(0.67-1.41)	0.89	1.10(0.88-1.38)	0.39	1.16(0.89-1.51)	0.27
Legumes	1.06(0.87-1.29)	0.55	1.18(0.79-1.74)	0.42	1.09(0.86-1.38)	0.49	0.93(0.70-1.24)	0.62
Daily fiber intake	0.99(0.86-1.15)	0.93	0.73(0.52-1.01)	0.06	0.98(0.83-1.16)	0.82	1.03(0.84-1.26)	0.78
**DASS-Depression**								
Vegetables	1.30(0.85-1.98)	0.23	1.09(0.66-1.81)	0.74	**1.64(1.03-2.62)**	**0.03***	1.05(0.64-1.73)	0.84
Fruit	1.11(0.73-1.70)	0.62	1.17(0.71-1.94)	0.53	1.00(0.62-1.61)	0.99	0.84(0.51-1.38)	0.49
Cereals, Bread, whole grain	1.05(0.72-1.52)	0.79	1.37(0.86-2.18)	0.18	0.91(0.60-1.39)	0.67	0.88(0.57-1.37)	0.57
Nuts and seeds	0.95(0.75-1.19)	0.64	0.91(0.69-1.21)	0.53	0.94(0.72-1.24)	0.68	0.88(0.66-1.17)	0.38
Legumes	1.10(0.85-1.42)	0.46	1.28(0.94-1.74)	0.12	1.09(0.82-1.46)	0.55	0.99(0.73-1.35)	0.96
Daily fiber intake+	1.09(0.91-1.31)	0.36	1.07(0.86-1.33)	0.54	1.05(0.85-1.30)	0.63	1.06(0.85-1.32)	0.59
